# Venous hemodynamics in neurological disorders: an analytical review with hydrodynamic analysis

**DOI:** 10.1186/1741-7015-11-142

**Published:** 2013-05-31

**Authors:** Clive B Beggs

**Affiliations:** 1 Medical Biophysics Laboratory, School of Engineering, Design and Technology, University of Bradford, Bradford, West Yorkshire BD7 1DP, UK

**Keywords:** Venous hypertension, CCSVI, Multiple sclerosis, Leukoaraiosis, Normal-pressure hydrocephalus, Cerebral blood flow, Cerebrospinal fluid

## Abstract

Venous abnormalities contribute to the pathophysiology of several neurological conditions. This paper reviews the literature regarding venous abnormalities in multiple sclerosis (MS), leukoaraiosis, and normal-pressure hydrocephalus (NPH). The review is supplemented with hydrodynamic analysis to assess the effects on cerebrospinal fluid (CSF) dynamics and cerebral blood flow (CBF) of venous hypertension in general, and chronic cerebrospinal venous insufficiency (CCSVI) in particular.

CCSVI-like venous anomalies seem unlikely to account for reduced CBF in patients with MS, thus other mechanisms must be at work, which increase the hydraulic resistance of the cerebral vascular bed in MS. Similarly, hydrodynamic changes appear to be responsible for reduced CBF in leukoaraiosis. The hydrodynamic properties of the periventricular veins make these vessels particularly vulnerable to ischemia and plaque formation.

Venous hypertension in the dural sinuses can alter intracranial compliance. Consequently, venous hypertension may change the CSF dynamics, affecting the intracranial windkessel mechanism. MS and NPH appear to share some similar characteristics, with both conditions exhibiting increased CSF pulsatility in the aqueduct of Sylvius.

CCSVI appears to be a real phenomenon associated with MS, which causes venous hypertension in the dural sinuses. However, the role of CCSVI in the pathophysiology of MS remains unclear.

## Introduction

The cerebral venous system is often viewed simply as a series of collecting vessels channeling blood back to the heart, yet it also plays an important role in the intracranial hemodynamic/cerebrospinal fluid (CSF) regulatory system (hereafter simply referred to as the hydrodynamic regulatory system), a role that is often overlooked and that appears to influence both perfusion of the brain parenchyma [1,2] and the dynamics of the CSF system [3-5]. Although the physiological mechanisms associated with cerebral-venous outflow are poorly understood, abnormalities of the venous system have been implicated in a variety of neurological disorders, including multiple sclerosis (MS) [4,6-11], leukoaraiosis [3,12-16], vascular dementia [5,17], and normal-pressure hydrocephalus (NPH) [2,3]. This raises intriguing questions about the involvement of the venous system in these pathophysiologies. Furthermore, similarities between the intracranial hydrodynamic phenomena associated with these conditions suggest that common physiological mechanisms may be at work. This paper reviews the literature relating to the role of the venous system in MS, leukoaraiosis, and NPH, with the aim of better understanding the contribution of venous hemodynamics to these neurological disorders.

### Evidence of venous involvement

Despite having very different pathologies, MS, leukoaraiosis, and NPH all share some common characteristics. In all three conditions, cerebral blood flow (CBF) is reduced [18-21]. Both MS [6,10] and leukoaraiosis [13,14,22,23] are characterized by changes in the white matter (WM) in the periventricular region, and enlarged lateral ventricles are associated with both MS [24,25] and NPH [26,27]. Furthermore, some clinical characteristics are also shared. Gait disturbances [28-31], reduced cognitive ability [30,32,33], and loss of bladder control [30,34,35] have been reported for all three conditions. This suggests that the pathophysiology of these disparate conditions might share a common feature. Having said this, all three diseases display marked pathophysiological differences. For example, MS is an autoimmune disease, characterized by brain atrophy [36,37], and it is thought that this is primarily responsible for ventricular enlargement [24,25]. Although the ventricles are also enlarged in NPH, brain parenchymal atrophy is not evident [26], and a measure of ventricular normality can sometimes be restored by the surgical insertion of a shunt to drain away excess CSF [27,38,39]. Although MS and leukoaraiosis both exhibit periventricular WM changes, leukoaraiosis is thought to be one presentation of cerebral small-vessel disease [40], whereas MS is a chronic inflammatory demyelinating disease of the central nervous system (CNS) [41]. Consequently, MS therapies focus on preventing axonal demyelination and promoting remyelination [42,43], whereas vigorous treatment of cardiovascular risk factors has been advocated to prevent the development of leukoaraiosis, and to reduce the attendant risk of stroke and dementia [44].

#### Multiple sclerosis

Since the earliest years of research into MS, there has been suspicion that the venous system might be involved in its etiology, with Dawson [10], Putnam [6,11] and others [45-49] all implicating veins in the pathophysiology of the disease. MS plaques are often venocentric, and frequently form around the periventricular veins [6]. Dawson [10] reported that finger-like plaques form at the junction of the subependymal and medullary veins in the periventricular WM. Putnam and Adler [6], commenting on the appearance of these ‘Dawson’s fingers’, observed that the medullary veins were enclosed in a sleeve of plaque, and that, adjacent to the plaques, the veins were grossly distorted and distended. Others [45,50-52] have also shown that inflammatory lesions tend to form axially around veins in the WM, with Tallantyre *et al*. [53] finding 80% of MS lesions to be perivenous in nature. Kidd *et al*. [50] showed that lesions in the grey matter (GM) are also associated with veins, with the majority of cortical lesions arising within the territory of the principal vein, V_5_, whose course begins in the WM [54], and the remaining cortical lesions forming in the region drained by its branches or those of the superficial veins. Others have confirmed these observations, finding intracortical [55-57], leucocortical [55], and sub-cortical [52] lesions to be perivenous. However, the connection with the venous system has largely been ignored, with the focus of MS research turning instead towards the involvement of the immune system in the disease [58,59].

Recently, there has been renewed interest in studying vascular changes associated with MS [60-62]. This has been precipitated by the controversial finding that abnormalities in the extracranial venous system appear to be associated with the disease [4,7,8,63]. This condition, known as chronic cerebrospinal venous insufficiency (CCSVI), is characterized by multiple intraluminal stenotic malformations of the principal venous-drainage pathways, particularly in the internal jugular veins (IJVs) and the azygos vein, and has been shown to be associated with impaired blood flow from the brain to the heart in patients with MS [8], with the hydraulic resistance of the cerebral-venous drainage system being on average 63.5% greater in CCSVI-positive individuals [64]. CCSVI also appears to be associated with changes in the intracranial vasculature, with a strong correlation shown between CCSVI severity and decreased CBF in both the WM and GM of patients with MS [65]. In addition, Zivadinov *et al*. [9] reported a marked reduction in venous vasculature visibility (VVV) on susceptibility-weighted imaging (SWI) for cerebral veins of less than 0.3 mm diameter in patients with MS compared with controls, a phenomenon that is strongly statistically associated with CCSVI (*P* < 0.0001). This finding appears to corroborate the work of Ge *et al*. [66]. However, unlike Ge *et al*., who attributed the reduction in VVV to hypometabolic status in the brain parenchyma of patients with MS, Zivadinov *et al*. performed a pre-contrast and post-contrast SWI venography experiment, which indicated the reduction in VVV to be due to morphological changes in the cerebral veins of patients with MS. Indeed, such was the clear-cut nature of these venous changes that Beggs *et al*. [67] were able to distinguish between patients with MS and healthy controls with 100% accuracy using cerebral-venous data alone.

These findings reinforce a large body of evidence connecting MS with alterations in the cerebral vascular bed. Using tomography, a number of early investigators [68-71], found reduced CBF in the GM and WM of patients with MS. However, this work received little attention and it was not until the advent of magnetic resonance imaging (MRI) that interest was renewed [61]. Using dynamic susceptibility contrast-enhanced MRI, Law *et al*. [18], identified a 53.4% decrease in CBF throughout the normal-appearing white matter (NAWM) in patients with relapsing–remitting (RR) MS compared with controls. This was accompanied by a twofold increase in vascular mean transit time (MTT), and a 13.6% decrease in WM cerebral blood volume (CBV). Adhya *et al*. [21] studied tissue perfusion in the NAWM of patients with primary progressive MS, relapsing-remitting (RR) MS, and healthy controls. They also found CBF and CBV to be significantly decreased in all NAWM regions in both forms of MS compared with controls. Similarly, Ge *et al*. [19] found reduced CBF with significantly prolonged MTT in the NAWM to be a feature of MS. Varga *et al*. [20] reported blood flow to be particularly low in the periventricular region, with CBF in the NAWM in this region being significantly lower in patients with MS compared with controls. Interestingly, they also found CBF to be decreased in the sub-cortical normal-appearing grey matter in patients with RR MS, suggesting a continuum of decreased tissue perfusion, beginning in the WM and spreading to the GM as the disease progresses [60]. Collectively, these findings indicate that hypoperfusion of the WM is a consistent phenomenon in MS, whatever the disease subtype [61]. Several researchers have also found MS to be associated with reduced CBF in the GM. Rashid *et al*. [72] found hypoperfusion in several cortical areas of patients with RR and progressive MS. Investigating tissue perfusion in the thalamus, putamen, and caudate nuclei of patients with MS, Inglese *et al*. [73] found a decrease in CBF in the deep GM, the magnitude of which increased with the severity of the disease. These findings, along with those relating to the WM, suggest that MS is associated with systemic changes in blood flow through the cerebral vascular bed, something highlighted by Mancini *et al*. [74], who found the mean tissue-vein transit time to be 3.2 seconds in patients with MS, compared with only 2.9 seconds in healthy controls.

Venous hypertension in the dural sinuses is known to inhibit absorption of CSF through the arachnoid villi (AV) [75,76]. Zamboni *et al*. [4] reported reduced CSF net flow and increased CSF pulsatility in the aqueduct of Sylvius (AoS) in patients with MS, and found this to be strongly associated with CCSVI severity. Magnano *et al*. [77] also found MS to be strongly associated with increased aqueductal pulsatility and reduced CSF net flow. Although Magnano *et al*. did not specifically consider CCSVI, their findings are consistent with those of Zamboni *et al*., and suggest that venous hypertension may be a feature of MS. Abnormal CSF hydrodynamics have also been implicated in the formation of cortical lesions in MS. Sub-pial lesions, which appear not to be perivenous, cover extensive areas of the cortex, and extend from the surface into the brain [55]. They appear to be mediated by infiltrates, generated by inflammatory cells in the meninges or the CSF, which diffuse inwards from the surface of the brain [55,78]. Kutzelnigg *et al*. [79] found sub-pial demyelination to be most pronounced within deep invaginations of the cortex, and suggested that this reflected regional differences in CSF flow, with extensive demyelination occurring in areas of CSF stasis.

#### Leukoaraiosis

Leukoaraiosis is a radiological finding, characterized by WM hyperintensities in the periventricular region on T2-weighted MRI scans [80], which is associated with diverse clinical symptoms, including cognitive impairment [81], vascular dementia [82,83], gait disturbance [30], and enhanced risk for stroke [84]. Although leukoaraiosis is primarily a pathology associated with aging [83,85], it shares several similarities with MS [62,86,87]. Both diseases affect the WM and are associated with demyelination [13,82]. In a similar manner to MS, leukoaraiosis is characterized by WM morphological changes around the periventricular veins [13,14,22,23]. Although not fully understood, leukoaraiosis is thought to be associated with chronic cerebral ischemia [88]. In cases of hypoxic/ischemic injury, histological changes of the WM can range from coagulative necrosis and cavitation to non-specific tissue changes such as sponginess, patchy demyelination, and astrocytic proliferation [88]. Such changes are consistent with the lesions seen in patients with leukoaraiosis [89], suggesting that ischemia is closely associated with the condition [88]. In particular, leukoaraiosis is characterized by non-inflammatory collagenosis of the periventricular veins [13,22], resulting in thickening of the vessel walls and narrowing, or even occlusion, of the lumen [13]. Moody *et al*. [13] found a strong association between the probability of severe leukoaraiosis and periventricular venous collagenosis (PVC).

Mirroring the cerebral hemodynamics of MS, several researchers have reported leukoaraiosis to be associated with reduced CBF [23,83,90,91]. However, unlike MS, a strong epidemiological link exists between leukoaraiosis and cerebrovascular disease [92-94]. Arterial hypertension and cardiac disease are also risk factors that are frequently associated with leukoaraiosis [88], and these are thought to induce arteriolosclerotic changes in the arteries and arterioles of the WM, replacing the smooth muscle cells by fibro-hyaline material, causing thickening of the vessel walls and narrowing of the vascular lumen [95]. Indeed, arteriolosclerosis is often present within areas of leukoaraiosis [96,97]. Furthermore, the arterioles supplying the deep WM, which are some of the longest in the brain, frequently become tortuous with aging [23,98-100], with the result that there is a trend towards increased tortuosity in individuals with leukoaraiosis [23]. This tortuosity usually begins abruptly as the arteriole passes from the cortex into the WM [23], and greatly increases the vessel length. Given that this will increase the hydraulic resistance of the arterioles [99], it will tend to inhibit the blood flow to the deep WM. It is therefore perhaps not surprising that the periventricular veins, being a ‘distal irrigation field’ [88], are prone to ischemic damage under conditions of moderate deficit in blood flow.

Further evidence linking leukoaraiosis with altered venous hemodynamics comes from a series of studies by Chung and co-workers [15,16,101], who investigated jugular venous reflux (JVR) (that is, retrograde flow in the IJVs) in older individuals. They found JVR to be a phenomenon that increased with age, and concluded that it was associated with more severe age-related WM changes (leukoaraiosis) [16]. In particular, they found that the IJV lumen cross-sectional area increased with age [101], which suggests dilation of the veins due to increased venous pressure and reduced flow velocity. Chung *et al*. [101] suggested that if the venous hypertension exceeds the ability of the dilation to compensate for the additional pressure, then it would compromise the competence of the jugular venous valves, with the result that the direction of venous flow could be reversed. They further hypothesized that this ‘chronic or long-term episodic elevated cerebral-venous pressure might cause cerebral venule hypertension, resulting in… reduce[d] CBF since elevated cerebral venule pressure would lower cerebral perfusion pressure’ [15].

In a series of studies, Bateman and co-workers investigated altered venous hemodynamics in a variety of neurological conditions [2,3,5,17,102,103]. In particular, they investigated pulsatile blood flow in leukoaraiosis [3] and vascular dementia [5]. In both conditions, they found venous pulsatility to be greatly increased in the straight sinus compared with healthy controls, implying that in individuals with leukoaraiosis and vascular dementia, the blood flow through the WM is highly pulsatile. Given that blood flow through the cerebral vascular bed is generally non-pulsatile in healthy young adults [3,104], Bateman’s findings imply marked changes in hemodynamic behavior in individuals with leukoaraiosis and vascular dementia, something that will induce transient shear stresses on the endothelia. Given that vessels experiencing highly oscillatory flows also seem to be at high risk of developing arteriosclerosis [105], it is perhaps not surprising that leukoaraiosis is associated with morphological changes in the WM vasculature [13,14,22,23]. Bateman hypothesized that the increased pulsatility exhibited by the CBF was a direct consequence of a dysfunctional windkessel mechanism [3,5], implying profound alterations in the dynamics of the CSF system. Indeed, Bateman calculated the CSF pulse volume in severe cases of leukoaraiosis to be 46% greater than that in healthy controls [3]. Furthermore, he found that the CSF dynamics associated with leukoaraiosis delayed the egress of blood from the cortical veins into the superior sagittal sinus (SSS), inducing a complex pulse wave, which propagated backwards towards the capillaries of the cortex [3].

#### Normal-pressure hydrocephalus

NPH occurs when there is an abnormal accumulation of CSF in the ventricles, causing them to become enlarged [27], but with little or no increase in intracranial pressure (ICP). Most adults with the condition experience an ICP that is not unusually high, being generally less than 15 mmHg [106,107]. NPH is characterized by gait disturbance, urinary incontinence, and dementia [108]. Although its pathophysiology is poorly understood, NPH has traditionally been thought to be a form of communicating hydrocephalus, characterized by poor absorption of CSF into the SSS due to defective AV [109]. However, evidence supporting this opinion is lacking [109], and several commentators have suggested alternative theories [2,102,110-112]. In particular, there is growing evidence that reduced intracranial compliance [2,102,113,114], induced by venous hypertension, might be involved in the pathophysiology of NPH [2,102,115,116], although this opinion is disputed by others [26,117,118]. Bateman [102] found the arteriovenous delay (AVD), a general marker of intracranial compliance, to be 53% lower in patients with NPH compared with healthy controls. A similar reduction in AVD in patients with NPH was reported in a subsequent study [2]. Mase *et al*. [114] independently confirmed this finding, showing a 64% reduction in intracranial compliance in patients with NPH compared with controls. The fact that an AVD exists at all indicates the presence of compressible material within the intracranial space, which is deformed when the systolic arterial pulse enters the cranium. With respect to this, the cerebral veins are a likely candidate [115,116]. Approximately 70% of intracranial blood volume is located within the venous compartment, much of it in thin-walled veins that readily collapse under small changes in transmural pressure. Given that the intracranial veins, particularly those of the superficial venous system, are much more compliant than the arterial vessels, it has been suggested that the change in intracranial compliance seen in patients with NPH may be associated with venous hypertension [2]. In patients with NPH, cortical-vein compliance is significantly reduced [102]; however, following shunt surgery, compliance greatly increases, suggesting that the compliance changes associated with these veins are functional and not structural [2,102]. NPH has been shown to be associated with venous hypertension in the SSS [119]. It is therefore plausible that hypertension in the SSS might increase the pressure in the cortical veins, with the result that the functional compliance of these vessels is reduced [2]. Furthermore, venous hypertension in the SSS would tend to reduce the compliance of the AV, and this, together with reduced cortical-vein compliance, might account for the reduction in AVD seen in individuals with NHP.

CBF has been found to be lower in patients with NPH than in normal controls [120-123]. This is generally thought to be associated with the formation of ischemic lesions, particularly in the deep WM [118,122,124], implying that regional differences in CBF might exist in individuals with NPH. Momjian *et al*. [122] found the distribution of regional CBF in the WM to be different in patients with NPH compared with healthy controls, with a more pronounced CBF reduction adjacent to the lateral ventricles, and a logarithmic normalization occurring with distance from the ventricles. These findings built on an earlier study by Owler *et al*. [121], who reported NPH to be associated with a marked reduction in mean CBF in the deep GM. Momjian *et al*. [122] attributed these phenomena to a combination of factors, including cerebral small-vessel disease; tissue distortion, and reversal of CSF and interstitial fluid flow, resulting in reduced cerebral perfusion pressure (CPP) near the ventricles and resultant ischemia. However, this interpretation was challenged by Bateman [102], who found blood flow in the straight sinus, which serves the periventricular region, to be unchanged in patients with NPH compared with controls. Having said this, Bateman also reported 27% less drainage from the SSS in patients with NHP compared with healthy controls. Although Bateman’s findings concerning the blood flow through the deep venous system are difficult to explain, those relating to the superficial venous system, might help to explain the formation of cortical infarcts in patients with NPH reported by Tullberg [124].

A number of researchers have reported marked alterations in CSF dynamics in NPH, with CSF pulsatility in the AoS found to be markedly greater in patients with NPH compared with controls [112,125-129]. This mirrors the findings of Magnano *et al*. [77], who found a similar phenomenon in patients with MS. By contrast, the cervical CSF pulse was either unchanged [112] or actually reduced in individuals with NPH compared with controls [126]. Although the reasons for this apparent paradox are difficult to explain, it suggests that biomechanical changes occur with NPH, which alter both intracranial compliance and pulsatility of the cerebral venous and arterial blood flows. NPH also appears to be associated with significantly reduced CSF resorption into the SSS through the AV [26,130], which is a finding consistent with venous hypertension in the dural sinuses. Drainage of CSF into the dural venous sinuses requires a pressure gradient between the sub-arachnoid space (SAS) and the SSS of about 5 to 7 mmHg [131,132]. If the pressure in the SSS is increased, then either the ICP must also increase to facilitate CSF absorption through the AV [117], or alternatively the CSF must be absorbed elsewhere in the intracranial space. Given that ICP does not substantially increase in individuals with NPH, this indicates that CSF is being resorbed elsewhere [124]. Bateman [102] suggested that CSF resorption is likely to occur in the sub-ependymal brain parenchyma. Ventricular reflux of fluid has been shown to be a characteristic of communicating hydrocephalus [133,134], with the periventricular tissue characterized by disruption of the ependyma, and by edema, neuronal degeneration, and ischemia [124]. Although the hydrodynamics associated with ventricular reflux are poorly understood, it may be that reduced CSF absorption by the AV in individuals with NPH at least partly explains the increase in aqueductal CSF pulsatility that is associated with the condition [133].

### Mass transfer and spatial proximity

Although there are clear differences in the pathologies of MS, leukoaraiosis and NPH, there are also striking similarities. All three are characterized by: 1) WM changes in the periventricular region; and 2) reduced CBF. The lesions associated with both MS and leukoaraiosis tend to be perivenous in nature, and the changes in CSF dynamics associated with NPH and MS also reveal similarities. This raises intriguing questions as to why these similarities exist. Are there some underlying physical mechanisms that are common to all these conditions?

The proximity of immune-cell aggregations to the vasculature is a hallmark of MS [135]. Whereas much attention has been paid to the biological mechanisms involved in the formation of MS plaques, the implications associated with their spatial arrangement have largely been ignored. Why do MS plaques form next to veins rather than capillaries, and why do they consistently occur in some places and not others? In nature, when a process is truly random, events tend to be widely distributed, with no underlying pattern. Conversely, if there is an underlying phenomenon, then the events will tend to cluster in both time and space. The fact that MS plaques consistently form around the periventricular [136] and cortical [50] veins indicates that the latter, rather than the former process must be taking place. If this were not so, then lesions would be randomly distributed throughout the brain parenchyma. From this, it can be concluded that in MS some unknown, but consistent, phenomenon is at work, which causes plaques to form around certain cerebral veins. Although historically considered a disease primarily affecting the WM, it is now known that cortical demyelination is common in MS and more extensive than previously appreciated [78,137]. Although it has been reported that many intra-cortical and sub-cortical lesions are perivenous in nature [50,52,55-57], it is not known why this is so. However, the fact that MS plaques form in the vicinity of veins in both the deep and superficial systems suggests that the pathophysiological mechanisms at work are extensive and not confined to a focal region.

Another universal principle found in nature is that of mass transfer. In simple terms, in order for matter to move from one place to another, it must be transported by some mechanism. In biology, the transport of cells and chemicals generally occurs either by: diffusion, by active transport (in the case of ion transport across the cell membrane), or through transport in a bulk fluid such as blood. If diffusion or active transport are the mechanisms at work, then there is a tendency towards higher concentrations of the transported substance near its source and lower concentrations further away. If this simple logic is applied to the formation of perivenous MS lesions, it would suggest that the plaque formation emanates from the blood vessels, rather than the other way round. Indeed, the current thinking appears to support this, suggesting that in MS, plaque formation is precipitated by breaching of the blood–brain barrier (BBB) [51,138,139]. If the diffusion principle is applied to the observation by Momjian *et al*. [122] that in patients with NPH the CBF steadily increases the further away from the lateral ventricles, then it suggests that the unknown factor inhibiting blood flow is emanating from the lateral ventricles, which suggests that ventricular fluid reflux might be involved.

The mass transport associated with bulk fluids also appears to offer insights into the spatial arrangement of ischemic WM changes, such as those found in leukoaraiosis. Considering oxygen transport in the blood through the cerebral vascular bed, the law of mass transport dictates that as oxygen is supplied to the brain parenchyma, so the oxygen levels in the blood will decrease. Consequently, the oxygen tension in the cerebral arteries will be higher than that in the cerebral veins. Under normal circumstances, this should not cause any problems, but when CBF is greatly impaired, as in both leukoaraiosis [23,83,90,91] and MS [18-21], then this might create pathogenic conditions in the distal veins. If the oxygen consumption of the endothelia and brain parenchyma surrounding the arterioles and capillary bed is not downregulated, then the oxygen tension in the veins might become so low that ischemic damage could occur in these vessels. With respect to this, the periventricular WM, being at the distal end of the circuit [88], appears to be particularly vulnerable to ischemic damage when blood flow is reduced. If ischemic damage due to hypoperfusion is involved in the formation of MS lesions, as some have suggested [60,61], then this might explain why plaques tend to form around the veins, rather than the capillaries and arterioles.

### Hypoxia

There is increasing evidence that hypoxia-like metabolic injury may be a pathogenic component in the formation of MS lesions [62,86]. Wakefield *et al*. [140] found morphological changes in the venous endothelia, which progressed to occlusive vascular inflammation. They proposed that these changes were the precursor to lesion formation, and suggested that demyelination in MS may have an ischemic basis. Aboul-Enein and Lassmann [141] reported similarities between the tissue injury found in inflammatory brain lesions and that found in hypoxic conditions of the CNS. Ge *et al*. [142] identified subtle venous wall signal changes in small MS lesions, which they interpreted as early-stage vascular changes. These changes may be the result of early ischemic injury, marking the beginning of trans-endothelial migration of vascular inflammatory cells, before any apparent BBB breakdown. Further evidence that focal inflammatory BBB leakage may not be the initiating event in MS plaque formation comes from Werring *et al*. [143], who measured random motion of water molecules (apparent diffusion coefficient; ADC) in the NAWM of patients with MS. They found that the formation of lesions was preceded by subtle progressive alterations in tissue integrity. Similarly, Wuerfel *et al*. [144] found that changes in perfusion parameters (CBF, CBV and MTT) were detectable not only prior to BBB breakdown, but also prior to increases in the ADC. They concluded that in MS, inflammation is accompanied by altered local perfusion, which can be detected prior to permeability of the BBB. Commenting on this, D’haeseleer *et al*. [60] concluded that ‘focal ischaemia might play a part in the development of a subcategory of focal MS lesions’. Lochhead *et al*. [145], using a rat model, showed that hypoxia followed by re-oxygenation altered the conformation of the occlusion in the tight junctions between the endothelial cells, resulting in increased BBB permeability. In doing so, they confirmed the findings of earlier studies undertaken by the same team [146,147]. The earliest detectable event in the development of WM lesions is thought to be an increase in the permeability of the BBB [51], followed by inflammation and demyelination. Others have implicated tight-junction abnormalities in increased BBB permeability and lesion formation in MS [139,148,149].

Several researchers have found similarities between leukoaraiosis and MS [60,61]. Leukoaraiosis is characterized by periventricular hyperintensities [80] and reduced CBF in the WM [150]. Its clinical symptoms include cognitive features that are similar to those associated with MS [151]. Graumann *et al*. [152], investigating gene expression in the NAWM of patients with secondary progressive MS and healthy controls, showed that the patients with MS exhibited consistent differences in the expression of hypoxia-inducible factor (HIF)-1a compared with controls. Similar upregulation of HIF-1a in cerebral WM was found by Fernando *et al*. [153] to be associated with leukoaraiosis, which they attributed to WM hypoperfusion. Leukoaraiosis is associated with significantly decreased CBF in the deep WM [150], and it is thought that ischemia, resulting from poor perfusion, is a major contributing factor [12,13,22]. The condition is characterized by non-inflammatory PVC, resulting in thickening of the vessel walls and narrowing of the deep cerebral veins [13,22], which will inevitably increase the hydraulic resistance of these pathways. Although lumenal narrowing of the periventricular veins has not been reported in patients with MS, Putnam and Adler [6] reported that the periventricular MS plaques resulted in gross distension of the medullary veins upstream of the lesions, suggesting that venous stenosis is occurring. This would inevitably increase the hydraulic resistance of these vessels and promote hypoperfusion. Given that the perfusion pressure, which promotes blood flow, is relatively low in the periventricular veins, the WM in this region is particularly sensitive to fluctuations in total CBF [154]. Any increase in the hydraulic resistance of the periventricular veins might cause shunting of blood away from these vessels [22,155], an action that would also tend to promote ischemia.

### Venous architecture

So why should some regions of the brain be more vulnerable than others to damage? Perhaps the architecture of the cerebral-venous system provides some clues? While the distal venous regions may be prone to hypoxic stress, the spatial arrangement of the veins may also contribute to their vulnerability. Evidence in support this opinion comes from Schlesinger [155], who forced hot carmine–gelatin solution, under high pressure, into the vein of Galen in human cadaver brains. The extravasations that were produced, chiefly in the region of the angle of the lateral ventricle, ‘closely resembled the distribution and shape of plaques in advanced cases of MS’. From this, Schlesinger concluded that: ‘it seems possible that the plaques may only be found in this area of the ventricular wall because they have a definite topographical relationship to the veins which are crowded together in the region of the lateral ventricular angle.’ Although the physiological implications of Schlesinger’s experiment are debatable, his results are clear and unambiguous from a fluid-mechanics point of view. When the gelatin solution was forced into the deep venous system, it divided and flowed up the two internal cerebral veins, so that both hemispheres of the brain were affected. Furthermore, the fluid flowed relatively easily until it came to the junction between the medullary and sub-ependymal veins, where the resistance was so great that the pressure built up to such an extent that the fluid burst through the vessel wall. The experiment therefore indicates two things: first, that the junction between the medullary and sub-ependymal veins has a much higher resistance to fluid flow than the downstream veins; and second, that the vessel walls at this junction are susceptible to rupture if the pressure gets too high.

The finding that the junction between the medullary and sub-ependymal veins has a high resistance to fluid flow is no surprise. The sub-ependymal veins are collecting vessels, which receive venous blood from a large number of the smaller medullary veins that enter the sub-ependymal veins at approximately 90 degrees. From a fluid-mechanics point of view, this is not a very streamlined configuration, and will result in relatively large pressure drop across this junction. Any stenosis at this junction would therefore greatly increase its resistance, possibly leading to distension of the upstream medullary veins, as Putnam and Adler reported [6]. Consequently, the periventricular veins share characteristics normally associated with developmental venous anomalies (DVAs). DVAs are a venous confluence in which a single collecting vessel drains an abnormally large venous territory, resulting in a relative volume overload. This anatomic configuration, as San Millán Ruíz *et al*. [156] pointed out, is similar to that encountered in the periventricular region. In addition, DVAs have been shown to have thickened walls [156], similar to those associated with PVC [13,22], with stenosis of the collecting vein reported in 13.1% of patients [156]. Stenosis of this kind invariably increases the hydraulic resistance of the vein, so that the upstream pressure is greatly increased, as was shown by Dillon [157], who measured a 15 mmHg pressure gradient across a stenosis of the collecting vein of a DVA in one patient.

Unlike the deep venous system, the superficial system has thin-walled cortical bridging veins that traverse the SAS. Blood flow through these compliant vessels is controlled by sphincters, which regulate discharge into the SSS [158,159]. This means that these vessels possess characteristics similar to those of a Starling resistor [160-163], and these collapse, occluding the blood flow, when the transmural pressure reaches a certain threshold [164]. The cortical bridging veins are very sensitive to small changes in transmural pressure. Indeed, because they are required to ‘open’ and ‘close’ to regulate blood flow from the cortex, the cortical venous pressure is only about 2 to 5 mmHg higher than the ICP [164]. This means that small changes in ICP or venous pressure can have a substantial effect on the behavior of blood flow from the cortex. Indeed, it has been estimated that a change of as little as 1.5 mmHg in the difference between ICP and the pressure in the bridging veins could be responsible for the difference between severe hyperemia (CBF = 1000 ml/min) to serve ischemia (CBF = 300 ml/min) [164]. Given that MS may be associated with venous hypertension in the dural sinuses of greater than 2 mmHg [165], it can be hypothesized that this could have a profound effect on blood flow in the cortex. Although it is difficult to say how this might influence hemodynamic behavior in the cortex, it is notable that Kidd *et al*. [50] found GM lesions in patients with MS to be exclusively located adjacent to cortical veins. Drawing an analogy with WM lesions, they stated; ‘We have been able to show that there is a clear relationship between the site and characteristics of cortical lesions and the five different types of cortical vein, just as Dawson’s ‘fingers’ arise adjacent to veins in periventricular WM’ [50].

### Cerebrospinal fluid dynamics and venous hypertension

**Figure 1:**
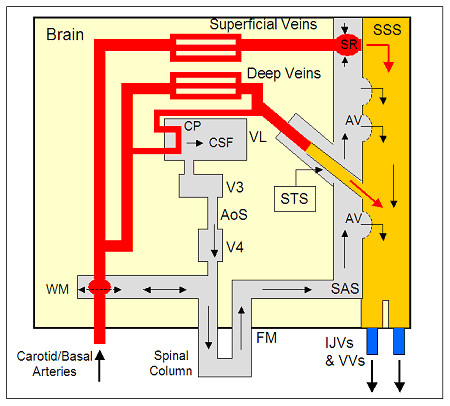
**Hydrodynamic model of the brain, showing the interactions between the arterial and venous blood flows and the cerebrospinal fluid (CSF).** SSS, superior sagittal sinus; STS, straight sinus; SAS, sub-arachnoid space; AV, arachnoid villi; CP, choroid plexus; FM, foramen magnum; WM, windkessel mechanism; SR, Starling resistor; VL, lateral ventricle; V3, third ventricle; V4, fourth ventricle; AoS, aqueduct of Sylvius; IJV, internal jugular vein; VV, vertebral veins.

MS, leukoaraiosis, and NPH all appear, to a greater or lesser extent, to be associated with marked changes in the dynamics of the intracranial CSF system. This suggests that these diseases might be associated with alterations in the intracranial hydrodynamic regulatory system, which controls the volume and pulsatility of the blood in the cerebral vascular bed [3,166,167]. The interactions between the CBF and CSF are illustrated in Figure 1, which shows an idealized model of the principal intracranial fluid pathways. From this, it can be seen that there is a bulk flow of CSF from the choroid plexus to the SSS, via the AV, driven by the pressure gradient between the two. There is also a complex windkessel mechanism that ensures Monro-Kellie homeostasis, which compensates for transient increases in CBV by pushing CSF out of the cranium [168]. This sophisticated windkessel mechanism uses the CSF to dampen the arterial pulse and to ensure, in healthy young adults, the smooth flow of blood through the capillary bed [5]. The energy from the arterial pulse is transferred to the CSF, which pulses backwards and forwards across the foramen magnum. The blood flow through the cerebral capillary bed is normally smooth and free from a pulse, but by the time it reaches the venous sinuses, it once again exhibits pulsatile characteristics [3,169]. This suggests that energy transferred from the arterial pulse to the CSF is in turn transferred back to the venous-discharge flow.

**Figure 2:**
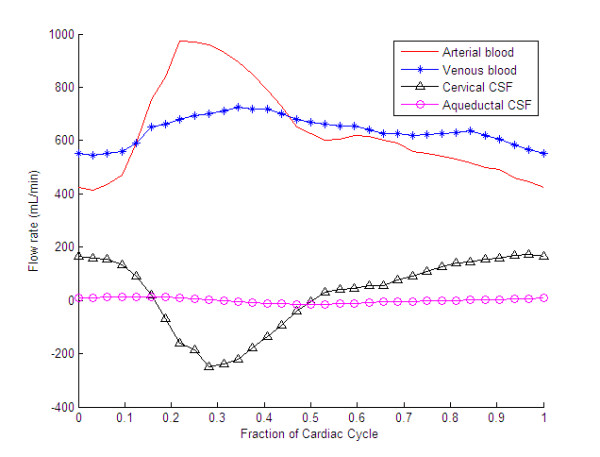
**Transient intracranial blood and cerebrospinal fluid (CSF) flow rates over the cardiac cycle in a healthy individual.** The figure is based on data published by Ambark *et al*. [169].

The various pulses associated with the intracranial hydrodynamic system are illustrated in Figure 2, which shows the transient flow rates of the arterial, venous, and CSF flows in and out of the cranium in a healthy individual [169]. From this, it can be seen that the system is driven by the arterial pulse, which, as it enters the cranium, greatly increases the volume of blood in the pial arteries during systole [1]. Peak CSF flow through the foramen magnum occurs shortly after the arterial peak, which is in turn followed in late systole by a peak in the venous blood flow leaving the cranium. This movement reflects the transfer of kinetic energy from the arterial to the venous pulse via the CSF system. By comparison, the aqueductal CSF pulse, which is not well understood, is much smaller than, and out of phase with, the cervical CSF pulse. In theory, according to the Monro-Kellie doctrine, at any point in time, the volume of CSF leaving the cranium should be equal to the volume difference between the arterial blood entering the cranium and the venous blood leaving it [170]. In reality, however, the Monro-Kellie doctrine is only approximately true [170], and compliance within the intracranial space, together with inertial forces associated with the CSF fluid column, ensure that small transient imbalances occur between the fluid volumes entering and leaving the cranium.

Close inspection of Figure 2 reveals an interesting and poorly understood phenomenon. During diastole, when the excess arterial blood stored by the windkessel mechanism is discharged back into the cerebral vascular bed, the venous blood-flow rate leaving the cranium actually decreases. This implies that during this period, the volume of venous blood in the cranium must be steadily increasing, only to be ejected during systole. Although the physiology associated with this mechanism is not well understood, it seems likely that much of this blood is stored during diastole in the cortical bridging veins that transverse the SAS. Discharge from these veins is controlled by regulatory sphincters [158,159]. Constriction of these sphincters results in an increase in the transmural pressure of the bridging veins, causing them to engorge and ‘puff out’, before periodically discharging into the SSS. Thus, these sphincters, in effect, create Starling resistors, whose characteristics are wholly governed by the respective venous and CSF pressures. Under normal circumstances, this Starling resistor interacts with the CSF pulse in such a way as to ensure the correct flow of blood into the SSS. However, if the venous pressure in the SSS should increase, due to partial occlusion of the cerebral-drainage pathways, then this would change the functional compliance of both the AV and the cortical bridging veins [2,102,115,116]. Under normal circumstances, the compliant AV dampen the systolic CSF pressure wave as the wave travels along the SAS, thus reducing the pressure that it exerts on the cortical bridging veins. Venous hypertension in the dural sinuses is therefore likely to reduce the time duration between arterial initiation of the CSF pulse and impingement of that pulse on the cortical bridging veins. Furthermore, it is likely to increase the intensity of the CSF pressure wave impinging on the bridging veins, so that the venous blood is expelled more rapidly into the SSS. Consequently, a reduction in the time duration between the arterial and venous peaks would be expected, which is exactly what Bateman found in patients with NPH [2,102]. However, the fluid mechanics associated with this mechanism are complex and it is difficult to predict how the system would behave under different circumstances. For example, if the hydraulic resistance of the cerebral-venous drainage system is greatly increased, as Beggs *et al*. [64] reported in patients with MS, then rapid discharging of the contents of the cortical veins might lead to a transient increase in pressure in the SSS. How this would behave in practice is a matter of conjecture. It might result in a complex reverse-pressure wave traveling down the cortical veins, as Bateman observed in an individual with leukoaraiosis [3], or alternatively, it could result in venous reflux traveling up the straight sinus, as has been suggested by Schelling [46].

Although the precise behavior of the intracranial hydrodynamic system under conditions of venous hypertension is unknown, there is evidence that occlusion of the venous-drainage pathways causes blood to accumulate within the cranium. In an experiment involving healthy subjects, Kitano *et al*. [171] showed that compression of the IJVs could result in an increase in intracranial blood volume of 5 to 20%. Frydrychowski *et al*. [1] also performed bilateral compression of the IJVs on healthy individuals, and found that it caused a reduction in the width of the SAS, a finding consistent with increased CBV. Furthermore, these authors found that during compression of the IJVs, the pulsatility of the pial arteries traversing the SAS increased by 107%. This suggests that occlusion of the venous-drainage pathways reduces compliance of the intracranial space, impairing the windkessel mechanism, with the result that the blood entering the cerebral microvasculature becomes more pulsatile. Frydrychowski *et al*. [1] concluded that this mechanism potentially linked jugular-outflow insufficiency with arterial small-vessel cerebral disease. Their work seems to corroborate that of Bateman [5], who found vascular dementia to be associated with greatly increased pulsatility in both the basal venous and straight sinus flows.

### Chronic cerebrospinal venous insufficiency

In 2009, Zamboni *et al*. published a paper [7] linking a vascular syndrome, CCSVI, with MS. CCSVI is characterized by restricted venous outflow from the brain [8,64] due to occlusions, which can take several forms, including the presence of intra-luminal septa, membranes, and immobile valves, as well as segmentary hypoplasia of the veins [172]. CCSVI has proven to be a highly contentious issue [173,174], with a number researchers doubting its validity as a physiological phenomenon [173,175-179]. Notwithstanding this, biomechanically, CCSVI will tend to increase the venous pressure in the dural sinuses, which hypothetically could alter the dynamics of the intracranial CSF system and potentially influence CBF.

The results obtained by researchers for CCSVI have been very mixed. For example, some researchers found CCSVI-like venous anomalies to be strongly associated with MS [7,63,180-186], whereas others found no significant difference between the venous characteristics of healthy controls and patients with MS [173,176,187-190]. Furthermore, CCSVI-like abnormalities are not exclusive to MS, and have been found in lesser numbers in healthy controls [74,180,191] and in those with other neurological diseases [180]. However, a recent meta-analysis of the published evidence showed a positive association between CCSVI and MS, although poor reporting and marked heterogeneity between studies precluded any definitive conclusions [192].

One possible explanation for the discrepancies between studies is the echo color Doppler sonography (ECDS) frequently used to diagnose CCSVI. The floppiness of the vessels involved and the variability of the venous vasculature can lead to erroneous results if ECDS is not undertaken correctly [193-195]. In an attempt to avoid these difficulties, Zamboni *et al*. [8] developed a non-invasive strain-gauge cervical plethysmography technique for characterizing cerebral-venous drainage in patients with MS. In a blinded study involving healthy controls and patients with MS diagnosed with CCSVI, it was found that the hydraulic resistance of the extracranial venous system was on average 63.5% greater in those diagnosed with CCSVI compared with controls (*P* < 0.001) [64]. This corroborates the work of Monti *et al*. [196], who found reduced cerebral-venous outflow in the upright position to be strongly associated (*P* < 0.0001) with MS.

#### Chronic cerebrospinal venous insufficiency and cerebrospinal fluid flow

Previous work [8,64,196] strongly suggests that people diagnosed with CCSVI exhibit reduced cerebral-venous outflow, which in some unknown way, is linked to MS. So if CCSVI is a real phenomenon, what role might it play in pathophysiology of MS? Is it a symptom, or part of the etiology? Although it is not currently possible to definitively answer these questions, it is possible, by considering the effects that occlusion of the IJVs might have on the cerebral hydrodynamic system, to gain insights into what might be happening.

Figure 1 describes the principal fluid pathways and interactions that occur within the cranium, and is therefore useful as a tool with which to interpret cerebral hydrodynamic behavior. In fluid mechanics, the flow through any pipe or vessel is governed by the following equation, which is analogous to Ohm’s law in electrical engineering:

(1) $$Q=\frac{\mathrm{\Delta P}}{R}$$

where *Q* is the fluid flow rate (ml/min), *R* is the hydraulic resistance (mmHg.min/ml), and Δ*P* represents the pressure drop (pressure gradient; mmHg) between the two ends of the vessel. By applying equation 1 to the intracranial system in Figure 1, it is possible to make predictions as to how the system would behave if the IJVs become occluded.

**Figure 3:**
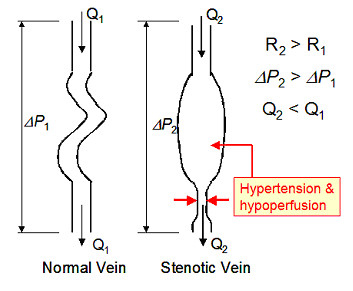
**Effect of stenosis on a vein.** Q_1_, blood-flow rate through normal vein; Q_2_, blood-flow rate through stenotic vein; R_1_, hydraulic resistance of normal vein; R_2_, hydraulic resistance of stenotic vein; ΔP_1_, pressure drop through normal vein; and ΔP_2_, pressure drop through stenotic vein.

One common feature of CCSVI is stenosis of one or both of the IJVs [7,197], which will tend to increase the hydraulic resistance of these pathways [64]. According to equation 1, any increase in the resistance due to partial occlusion of the IJVs will result in the two phenomena illustrated in Figure 3. Firstly, this increase in resistance will tend to reduce the flow rate of blood through the IJVs, and secondly, it will increase the pressure gradient through the vessel. Consequently, although hypoperfusion will occur, hypertension will also occur above the obstruction, as is evident by the distension of the IJV frequently seen in patients with CCSVI [165,197]. This increase in venous pressure will be transmitted up the vessels into the SSS, which has been shown in patients who exhibit thrombosis of the transverse sinuses [198,199]. Consequently, the increase in blood pressure in the SSS is likely to be the same order of magnitude as that in the IJV; that is, about 2.21 mmHg, according to measurements made by Zamboni *et al*. [165].

From Figure 1 it can be seen that the SSS acts as a collecting vessel for CSF from the SAS. The CSF bulk flow from the choroid plexus to the SSS via the AV, which in healthy individuals is around 3.3 to 5.5 mm^3^/beat (assuming 70 beats/min) [76], is very susceptible to changes in pressure [75]. In normal healthy individuals, the hydraulic resistance of the SAS is very low in comparison with the AV, which means that the outflow of CSF from the SAS to the SSS is almost entirely determined by the AV [76]. These open up when the pressure difference between the SAS and SSS is approximately 5 mmHg [75], allowing free absorption of CSF into the venous blood, a process that has been shown to be linear, with an average rate of 0.1031 ml/min/mmHg (0.0076 ml/min/mm H_2_0) [75]. Consequently, an increase of 2.21 mmHg in the SSS pressure would equate to a reduction in CSF bulk flow of about 3.26 mm^3^/beat (assuming 70 beats/min), which is close to the mean value of 3.4 mm^3^/beat reported by Magnano *et al*. [77] for reduction in CSF bulk flow in patients with MS compared with healthy controls, but somewhat lower than the mean difference of 11.86 mm^3^/beat reported by Zamboni *et al*. [4]. Collectively, these findings support the opinion that venous hypertension in the dural sinuses is a feature of CCSVI.

#### Chronic cerebrospinal venous insufficiency and cerebral blood flow

It is possible to gain an insight into the nature of the hemodynamic changes associated with MS, by undertaking simple hydrodynamic analysis of composite data published by Varga *et al*. [20]. These data are presented in Table 1, and represent measured blood flow in the periventricular WM.

**Table 1:** **Published blood-flow data in the periventricular white matter for healthy controls and patients with relapsing–remitting multiple sclerosis (RR MS)**[20]

**Source**	**Parameter**	**Healthy controls, mean ± SD**	**Patients with RR MS, mean ± SD**
Varga *et al*., 2009 [20]	CBF, ml/100 g/min	39.73 ± 5.0	33.53 ± 6.10
	CBV, ml/100 g	2.62 ± 0.60	2.40 ± 0.40
	MTT, s	3.94 ± 0.70	4.33 ± 0.50

Abbreviations: *CBF*, Cerebral blood flow; *CBV*, Cerebral blood volume; *MTT*, Mean transit time.

The data in Table 1 complies with the general relationship:

(2) $$\mathrm{CBF}=\frac{\mathrm{CBV}}{\mathrm{MTT}}$$

From the data it can be seen that in patients with MS, there is a general reduction in the volume of the vascular bed, which, if approximated to a series of parallel round tubes, equates to a mean reduction in cross-sectional area of the vessels of about 8.4% in patients with MS. According to Poiseuille’s Law:

(3) $$R\propto \frac{1}{r^{4}}$$

where R is the hydraulic resistance of the vessel (mmHg.min/ml) and r is the radius of the vessel (mm), it can be calculated that the 8.4% reduction in average cross-sectional area equates to an approximately 19.3% increase in hydraulic resistance. Given that the blood-flow rate is directly proportional to the hydraulic resistance, this means that the reduction in CBV seen in patients with MS, is more than enough to account for the 15.6% reduction in CBF reported by Varga *et al*.. According to equation 1, hypertension in the dural sinuses would tend to reduce the pressure gradient pushing the blood through the cerebral veins, which in turn would tend to inhibit blood flow. However, when we consider that the CPP is normally in the region of 70 to 90 mmHg, it is unlikely that venous hypertension of less than 5 mmHg, such as that associated with CCSVI, could account for the large reduction in WM CBF reported in patients with MS [18-21]. Hence, this suggests that the reduction in CBF in patients with MS is probably due to morphological changes in the cerebral vascular bed, rather than a straightforward reduction in perfusion pressure arising from raised pressure in the venous sinuses. However, this does not preclude the possibility that the reduction in CBF may also be due to changes in behavior of the Starling resistor associated with the cortical bridging veins.

Although the above analysis is somewhat simplistic, it does illustrate that cerebral vascular volumetric changes alone appear capable of accounting for the reduction in CBF in the periventricular NAWM in patients with MS. In addition, this finding mirrors those of researchers investigating: 1) reduced CBF [23,83,90,91]; and 2) WM morphological changes around the periventricular veins [13,14,22,23], in patients with leukoaraiosis. Although the above analysis assumes an overall reduction in CBV in patients with MS, this of course does not necessarily imply that all the vessels in the WM have uniformly narrowed, and the results reported by Putnam and Adler [6] regarding periventricular lesions in individuals with MS would suggest otherwise. However, in order to achieve an 8.4% overall reduction in CBV, systemic changes must be occurring in the WM vascular bed, rather than simply occlusions appearing at specific focal points. The finding by Varga *et al*. [20] of a 10% increase in MTT in patients with MS closely mirrors that of Mancini *et al*. [74], who reported an 8% increase in MTT. These results strongly suggest that the hydraulic resistance of the whole intracranial vascular circuit is significantly increased in patients with MS. Indeed, it may be the case that some vessels have disappeared completely, as reported by Zivadinov *et al*. [9], which would inevitably increase the hydraulic resistance of the cerebral vascular circuit.

Further evidence suggesting that occlusion of the cerebral-venous drainage pathways might not be responsible for reduced CBF in patients with MS comes from Moyer *et al*. [200], who compressed the jugular veins of patients with heart failure; Chai *et al*. [201], who performed jugular vein ligation in a swine model; and Bateman [202], who investigated idiopathic intracranial hypertension associated with venous outflow stenosis. All these researchers found occlusion of the venous pathways to be associated with CBF rates that were higher than normal. This counterintuitive finding could only be physically possible if the hydraulic resistance of the cerebral blood vessels were greatly reduced, suggesting that obstruction of the venous-drainage pathways results in vasodilatation and increased CBV. However, studies have found CBV to be reduced by 8.4 to 13.6% in patients with MS compared with healthy controls [18-20], suggesting that hyperemia may not in fact be a feature of this disease. Interestingly, Chai *et al*. [201] reported that cerebral oxygen consumption was significantly increased when venous ligation was applied, which suggests that venous hypertension might alter the metabolism of the brain. Although the effects of venous hypertension on cerebral metabolism are largely unexplored, it is known that the brain regulates blood flow according to its metabolic needs. Consequently, it is difficult to know the extent to which reduced CBF is initiated by venous hypertension or endothelial morphological changes, as opposed to downregulation of the metabolic activity of the brain.

## Conclusions

Although much research work has been undertaken into the contribution of venous abnormalities to various neurological conditions, there has generally been a lack of any hydrodynamic analysis to interpret the data collected. Without such analysis, it is possible to misinterpret results and come to potentially erroneous conclusions [174]. In the analytical review presented here, we have sought to redress this issue, and have been able to show that CCSVI-like anomalies in the extracranial venous system are unlikely to account for the reduction in CBF reported in patients with MS. Rather, our analysis suggests that other pathophysiological mechanisms must be a work, which are increasing the hydraulic resistance of the cerebral vascular bed in patients with MS. Similarly, changes in the cerebral microvasculature seem to be responsible for reduced CBF in leukoaraiosis. CBF in the WM is markedly reduced in both MS and leukoaraiosis, and in both conditions, lesions exhibit signs of ischemia, although to a lesser extent in the case of MS. Under conditions of hypoperfusion, the laws governing mass transfer indicate that the cerebral veins are more likely to be affected by hypoxic stress compared with the arterioles and capillaries, and this might, in part, explain why the plaques in MS tend to be perivenular in nature. With respect to this, the hydrodynamic properties of the periventricular veins appear to make these vessels particularly vulnerable to plaque formation.

Venous hypertension in the dural sinuses seems to be associated with marked changes in intracranial compliance. There is sound theoretical reason to believe that this will alter the dynamics of the intracranial CSF system, which in turn may affect the finely tuned intracranial windkessel mechanism. With respect to this, MS and NPH appear to share some similar characteristics. In particular, both conditions seem to be characterized by increased CSF pulsatility in the AoS.

Despite conflicting studies, there is increasing evidence that CCSVI is a real physiological phenomenon, and that it is in some way associated with MS. The evidence from CSF-related studies in patients with MS, and the hydrodynamic analysis presented here, suggests that CCSVI causes venous hypertension in the dural sinuses. However, the role that CCSVI might play in the pathophysiology of MS remains unclear, and more work is urgently needed to understand the clinical relevance of this condition.

## Abbreviations

ADC: Apparent diffusion coefficient; AoS: Aqueduct of Sylvius; AV: Arachnoid villi; AVD: Arteriovenous delay; BBB: Blood–brain barrier; CBF: Cerebral blood flow; CBV: Cerebral blood volume; CCSVI: Chronic cerebrospinal venous insufficiency; CNS: Central nervous system; CPP: Cerebral perfusion pressure; CSF: Cerebrospinal fluid; DVA: Developmental venous anomaly; ECDS: Echo color doppler sonography; GM: Grey matter; HIF: Hypoxia-inducible factor; ICP: Intracranial pressure; IJV: Internal jugular veins; JVR: Jugular venous reflux; MRI: Magnetic resonance imaging; MS: Multiple sclerosis; MTT: Mean transit time; NAWM: Normal-appearing white matter; NPH: Normal-pressure hydrocephalus; PVC: Periventricular venous collagenosis; RR: Relapsing–remitting; SAS: Sub-arachnoid space; SSS: Superior sagittal sinus; SWI: Susceptibility-weighted imaging; VVV: Venous vasculature visibility; WM: White matter.

## Competing interests

The author declares no conflicts of interest.

## Authors’ contributions

The study was conceived and undertaken by CBB, who also wrote the manuscript.

## Pre-publication history

The pre-publication history for this paper can be accessed here:

http://www.biomedcentral.com/1741-7015/11/142/prepub
